# Veterinary Medical Association of New York County

**Published:** 1896-05

**Authors:** Robert W. Ellis

**Affiliations:** Secretary


### VETERINARY MEDICAL ASSOCIATION OF NEW YORK COUNTY.

THE regular meeting was called to order at 9 P.M., April 7th, at the Academy of Medicine, by the Vice-President, Dr. Giffen, who expressed sincere regret at the circumstances which called him to fill the President's chair, Dr.

Huidekoper's serious illness. On roll-call the following members responded: Drs. Bretherton, E. C. Cattanach, J. S. Cattanach, J. L. Cattanach, Jr., Delaney, Dair, Ellis, Ferster, Finlay, Giffen, Gill, Hanson, Jackson, Johnson, Knott, Lambkin, Lellman, Machan, MacKellar, Neher, O'Shea, Ryder, Sherwood, Serling, and Turner (25).

Dr. Gill, Chairman Board of Censors, offered the resolution that whereas Dr. S. K. Johnson, by contracting with the Mutual Live-stock Benefit Insurance Company (?), has violated Article VII. of the Code of Ethics; therefore, be it resolved that the Board of Censors recommend to the Association that if the said Dr. S. K. Johnson does not show satisfactory evidence of having resigned and severed all connection, directly or indirectly, with the said insurance company on or before October 15, 1896, he be expelled from membership of this Association. The court records not having been produced in the case of Dr. Turner *vs.* Dr. Finlay, as was ordered at the last meeting, the charges against Dr. Finlay were dismissed and he was honorably acquitted. Owing to the absence of Dr. Glover from town, his case was laid over until the next meeting. Whereas, it has come to our knowledge that one or more members are directly or indirectly connected with live-stock insurance companies; therefore, be it resolved that all members so connected will either sever their connection with such companies or suffer expulsion. Moved and seconded that the report be accepted; carried. Moved and seconded that the secretary be authorized to notify any members who have any connection with insurance companies to sever such connection, according to report of Board of Censors; carried.

Committee on Legislation (Dr. O'Shea, Chairman) reported that the Cole Amendment Bill is to go into the Senate next week, and that the previous Cole Bill is killed. In reference to resolutions which were to be offered to the several city departments, and be introduced at Albany, the doctor reported that he had been advised that it was too late to pass that bill this year, and stated that association's counsel suggest that the committee see the heads of the various departments before introducing the bill at Albany, and thereby secure their mutual co-operation. Dr. Gill suggested to the meeting that the Civil-service Commission be conferred with on this matter, which suggestion was approved by the members. In reference to the Jury Bill, Dr. O'Shea reported that he had had Senator Sullivan's positive assurance that the bill would come out. Moved and seconded that the report be accepted; carried.

Dr. Neher reported a few cases of flatulent colic, in which he resorted to puncture of the stomach with good results; claiming that puncturing of the stomach would often save cases that could *not* be saved by puncturing in the flank; that eructations of gas were an indication for stomach puncture, and that point most resonant on percussion, just posterior to the cartilage of prolongation of the sternum, being the seat of puncture.

Dr. Hanson reported a case of fatty tumor weighing seven and a quarter pounds, which he removed from the sternal region just posterior to axilla in an Irish setter dog, which he had been called in consultation to see with Dr. Jackson.

A number of interesting cards issued by so-called veterinary surgeons were read by Dr. Neher, and referred to the Judiciary Committee for investigation. Moved and seconded that the secretary be authorized to communicate with the Secretary of the State Association and with Professor Law, of

Cornell University, enlisting their aid and co-operation with the County Association in securing a proper guardian of the interests of the veterinary profession of New York State at the Capital while the legislature is in session next year; carried. Moved and seconded that the secretary secure the list of registered men in New York County obtained by the Judiciary Committee, and have it printed; carried. Moved and seconded that the meeting adjourn; carried.

ROBERT W. ELLIS, D.V.S.,
Secretary.

